# From isolated data to integrated ecosystems: the artificial intelligence revolution in precision livestock farming

**DOI:** 10.1093/af/vfag010

**Published:** 2026-05-11

**Authors:** Victor E Cabrera, Mutian Niu

**Affiliations:** Department of Animal and Dairy Sciences, University of Wisconsin-Madison, Madison, WI 53706; Animal Nutrition, Institute of Agricultural Sciences, Department of Environmental Systems Science, ETH Zürich, 8092 Zürich, Switzerland

The global livestock and poultry farming systems are navigating an unprecedented paradigm shift. Faced with the compounding pressures of rising global protein demand, climate change, labor shortages, and increasing societal scrutiny regarding animal welfare, the agricultural sector is rapidly turning to digital solutions. Artificial intelligence (AI) is no longer a frontier concept confined to computer science laboratories; it is rapidly becoming the core engine driving the modernization of animal science, leading to novel on-farm applications.

This issue of *Animal Frontiers* offers a comprehensive exploration of this digital transformation. The eleven articles collected herein illuminate how the application of AI is evolving from the deployment of isolated, reactive tools to the creation of proactive, integrated knowledge systems (Figure 1).

**Figure 1.**
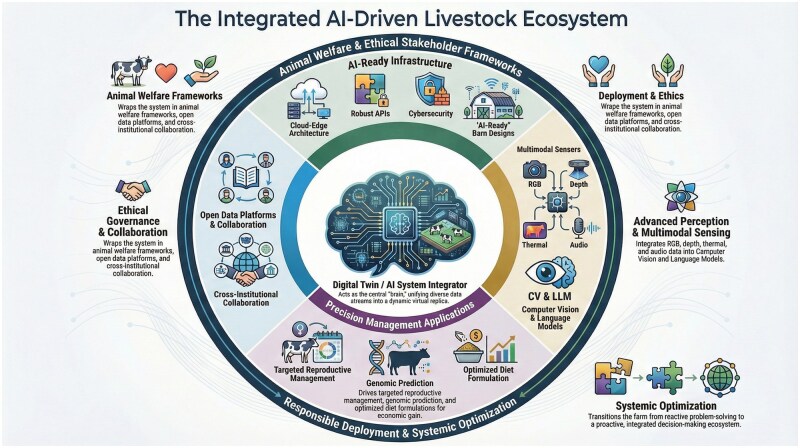
The integrated AI-driven livestock ecosystem. A conceptual framework illustrating the convergence of the four main themes explored in this special issue. At the core sits the AI System Integrator and Digital Twin (Niu et al., 2026), which continuously ingests data from the Infrastructure and Perception layer, encompassing “AI-ready” barn designs, cloud-edge collaborative architectures (Guo et al., 2026; Rosati, 2026), and multimodal sensors, including computer vision and audio (Ma et al., 2026; Paneru et al., 2026; Živković and Nasser, 2026). This data is processed to drive the precision management layer, enabling proactive, individualized interventions in nutrition (Bach, 2026), reproduction (Giordano and Laplacette, 2026), and genomic prediction (Morota et al., 2026). The entire ecosystem is encompassed by a governance and collaboration ring, ensuring cybersecurity, cross-institutional data sharing (Nasser and Kasper, 2026), and the integration of animal-centric ethical frameworks (Giersberg, 2026).

Together, these contributions chart the current capabilities of AI across species and disciplines—from computer vision and multimodal disease diagnosis to precision nutrition, genomic prediction, and reproductive management. Crucially, they also address the infrastructural, collaborative, and ethical barriers that must be overcome to fully realize AI’s potential.

## Rethinking Infrastructure and Systems Integration

To harness the power of AI, we must first address how data is collected, processed, and managed at the farm level. As Rosati (2026) points out, a fundamental error in current AI adoption is the “retrofit” approach—attempting to overlay intelligent tools onto traditional, pre-digital farm structures. Rosati (2026) argues for the holistic redesign of livestock environments to make them inherently “AI-ready,” which includes optimizing barn layouts for mechanization and sensor coverage, establishing rigorous cybersecurity protocols, and ensuring data interoperability among isolated farm systems.

The question of where and how this data is processed is equally critical. Guo et al. (2026) provide a systematic analysis of AI deployment pathways, comparing offline, on-premises, edge, cloud, and cloud-edge collaborative architectures. They conclude that while the choice of deployment depends on a farm’s specific operational context, such as capital, connectivity, and data privacy needs, the cloud-edge collaborative architecture emerges as the most promising paradigm because it balances the immediate, real-time responsiveness required at the edge with the massive computational and analytical power of the cloud (Guo et al., 2026).

Scaling up from infrastructure to epistemology, (Niu et al. 2026) articulate a profound conceptual shift: AI is evolving from a passive management tool into a system-level integrator and a co-producer of scientific knowledge. By fusing disparate data streams (e.g., nutrition, genetics, and environment), AI enables the creation of “digital twins”—dynamic virtual replicas of physical farms that allow for complex, proactive optimization (Niu et al., 2026). Furthermore, through advanced pattern recognition and generative capabilities, AI is positioned to autonomously generate testable scientific hypotheses, transforming the modern farm into a real-time living laboratory (Niu et al., 2026).

## The Ethical Imperative: Animals as Stakeholders

While optimizing infrastructure and algorithms is essential, the socio-ethical dimensions of AI deployment cannot be ignored. Giersberg (2026) delivers a vital critique of current AI ethics discourses, which have historically remained exclusively human-centered. Given that AI interventions intentionally and profoundly impact animal lives, altering their environments, interactions, and welfare, animals must be explicitly recognized as morally relevant stakeholders in AI development (Giersberg, 2026). Moving forward, multi-disciplinary discourses must integrate animal ethicists and welfare scientists to ensure that AI does not simply hyper-optimize productivity at the expense of animal well-being but genuinely enhances the “good life” of the animal (Giersberg, 2026).

## Advanced Perception: Multimodal Sensing and Computer Vision

A significant portion of this issue is dedicated to the remarkable advances in machine perception, particularly computer vision (CV) and multimodal sensing, which are replacing subjective human observation with continuous, objective monitoring.

In poultry production, Paneru et al. (2026) review the state of precision poultry farming, detailing how AI-driven tools, such as deep learning for behavior tracking, audio analysis for distress vocalizations, and robotics for floor egg collection, are enhancing welfare and operational efficiency. However, they also note that the true potential of these tools is hampered by a lack of standardized, open-access datasets and emphasize the need for rigorous ethical evaluation of automated systems (Paneru et al., 2026).

Ma et al. (2026) expand on this by exploring multimodal sensing across both poultry and livestock. Recognizing that single-sensor approaches are often vulnerable to the harsh, complex environments of commercial farms, they advocate for the fusion of diverse data streams—including RGB, depth, thermal imaging, and acoustic data. By transitioning from traditional database-driven expert systems to advanced, knowledge-driven frameworks powered by large language models and multi-agent systems, the authors illustrate a future of highly robust, intelligent disease diagnosis and decision support (Ma et al., 2026).

Focusing specifically on the swine industry, two articles address the practical implementation of computer vision. Živković and Nasser (2026) provide a hands-on practitioner’s comparison of two leading markerless pose estimation frameworks: DeepLabCut and SLEAP. Their evaluation reveals that while both tools achieve comparable high-level accuracy, they differ significantly in user experience, installation complexity, and multi-animal tracking capabilities (Živković and Nasser, 2026). They conclude that the choice of tool should be tailored to the specific technical skills and environmental setup of the research team (Živković and Nasser, 2026).

Despite these software advancements, a systemic bottleneck remains: data fragmentation. Nasser and Kasper (2026) issue a compelling call to action to address the scarcity of annotated, high-quality CV datasets in precision pig farming. They identify two core bottlenecks, annotation inefficiency and limited model generalizability, and propose an international, collaborative data and AI model platform (Nasser and Kasper, 2026). By pooling resources, utilizing auto-annotation, and standardizing metadata, such an initiative would dramatically lower entry barriers, enable reproducible scientific progress, and pave the way for robust foundation models tailored to livestock (Nasser and Kasper, 2026).

## Precision Management: Nutrition, Reproduction, and Genetics

The final thematic pillar of this issue focuses on the application of machine learning (ML) to optimize specific biological and management processes, moving away from static, population-level averages toward dynamic, individualized precision.

In dairy nutrition, Bach (2026) demonstrates how ML algorithms can revolutionize diet formulation and feeding management. Traditional factorial models rely on an “average cow” and struggle to account for complex, non-linear nutrient interactions and farm-specific variables. By integrating massive, granular datasets encompassing nutrition, genetics, health, and farm economics, ML models (such as gradient boost decision trees and reinforced learning) can accurately forecast immediate animal responses (Bach, 2026). This allows nutritionists to formulate diets and design grouping strategies that maximize income over feed cost rather than merely maximizing milk yield (Bach, 2026).

Similarly, Giordano and Laplacette (2026) explore the transformative opportunities of AI in dairy cattle reproduction. A full reproductive cycle involves complex physiological events—calving, uterine recovery, estrus, and pregnancy—that must be meticulously monitored. The authors detail how AI-driven tools, fed by deep phenotyping from wearable sensors and farm records, can highly accurately predict these reproductive events and estimate the overarching reproductive potential of individual cows (Giordano and Laplacette, 2026). By classifying cows into distinct fertility groups, AI enables targeted reproductive management strategies, optimizing interventions such as synchronization protocols and culling decisions to boost herd profitability (Giordano and Laplacette, 2026).

Finally, Morota et al. (2026) bridge the gap between AI and quantitative genetics. While AI, particularly deep learning (DL), has achieved massive success in digital phenotyping (e.g., using CV to extract biometric traits from images), its integration into genomic prediction remains challenging (Morota et al., 2026). The authors explain that while DL excels at capturing non-linear relationships in image or audio data, it currently struggles to consistently outperform traditional Genomic Best Linear Unbiased Prediction models when applied to tabular single nucleotide polymorphism datasets (Morota et al., 2026). Overcoming the specific spatial and structural complexities of genomic data will be the next great frontier for AI in animal breeding (Morota et al., 2026).

## Conclusion

The collective insights from this issue of *Animal Frontiers* make one thing abundantly clear: the future of livestock farming relies on integration. The industry must move beyond treating AI as a series of isolated, novelty “black boxes” and instead weave these technologies into the very fabric of agricultural infrastructure, management workflows, and genetic evaluations.

Achieving this requires a concerted, interdisciplinary effort. Data must be democratized through secure, open, and collaborative platforms to train robust, generalizable models. Furthermore, as we design the digital farms of tomorrow, we must embed ethical frameworks that prioritize animal welfare alongside environmental sustainability and economic viability. By embracing AI as both a management tool and a co-producer of knowledge, the animal science community is well-positioned to meet the profound agricultural challenges of the 21st century.

## Author Biography

**Dr. Mutian Niu** is an Assistant Professor of Animal Nutrition at ETH Zurich’s Institute of Agricultural Sciences, Department of Environmental Systems Science. His research advances sustainable livestock production by integrating hypothesis-driven experiments with data science, focusing on ruminant nutrition, nutritional physiology, greenhouse gas mitigation, nitrogen metabolism, heat stress alleviation, statistical modeling, precision livestock farming, and exhalomics/breathomics for non-invasive monitoring of animal health and emissions. Prior to ETH Zurich, Dr. Niu served as an Assistant Professor at the University of Pennsylvania’s School of Veterinary Medicine and as a Data Scientist at Farmer’s Business Network. He earned his Ph.D. in Animal Biology and M.S. in Statistics from the University of California, Davis; an M.S. in Animal Science from Pennsylvania State University; and a B.S. in Animal Science from China Agricultural University. Dr. Niu has secured > USD 3.4 million in grants, including projects on net-zero emissions pathways. He has authored >50publications in journals like the Annual Review of Animal Biosciences, Journal of Dairy Science, Global Change Biology, Animal, etc.. He serves as Co-Chair of the EAAP Precision Livestock Farming and AI for Animal Science Working Group, Guest Editor for Animal Frontiers, and a member of FAO LEAP and the Global Research Alliance on Agricultural Greenhouse Gases. His interdisciplinary approach bridges academia and industry to address global challenges in food security and climate change.

**Dr. Victor Cabrera** is a leading expert in data-driven decision support for dairy farm management, integrating applied research, interdisciplinary approaches, and participatory methods to develop practical, user-friendly tools that enhance farm profitability, environmental stewardship, and long-term sustainability. He leads the Dairy Brain project, a pioneering initiative in data integration and analytics for dairy farming, and actively contributes to the RuFaS project, which advances resilient and climate-smart agricultural systems. Dr. Cabrera and his research team have developed over 50 decision support tools, published more than 110 refereed articles and 12 book chapters, and his work has had a global impact through research and extension. He has presented at over 140 scientific sessions and delivered more than 400 extension talks across Wisconsin, the U.S., and internationally. Over the past 15 years, Dr. Cabrera has secured over $5.5 million in funding to support his research and extension initiatives. His contributions have been recognized with numerous prestigious awards, including the Vilas Associate Award, Excellence in International Activities, the DeLaval Dairy Extension Award from the American Dairy Science Association, the Vilas Faculty Mid-Career Investigator Award, and several other distinctions from UW-Madison, the Wisconsin Association of County Agricultural Agents, the University of Florida, and the American Dairy Science Association.
